# Supplementary material for: Genetic and epigenetic features of bilateral Wilms tumor predisposition in patients from the Children’s Oncology Group AREN18B5-Q
Source: Nat Commun. 2023 Dec 18;14:8006. doi: 10.1038/s41467-023-43730-0 (PMC10728430; doi:10.1038/s41467-023-43730-0)
Supplement: Supplementary file 1 — Supplementary Information [file 41467_2023_43730_MOESM1_ESM.pdf]

Supplementary File

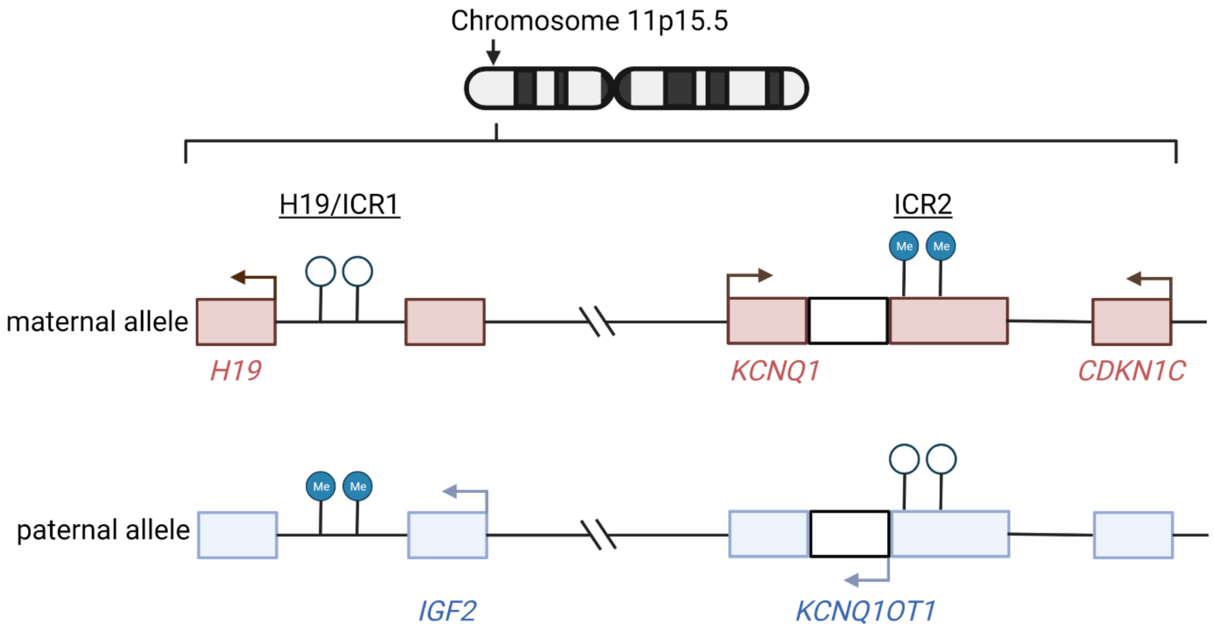

Supplementary Figure 1. Mechanisms of dysregulated imprinting at chromosome 11p15.5. Chromosome 11p15.5 houses a cluster of imprinted genes (including *IGF2, KCNQ1, CDKN1C*) and noncoding RNAs (including *H19*, *KCNQ1OT1*) that are either expressed only from the maternal or paternal allele. The expression of these genes is regulated by differential methylation at two imprinting control regions (ICR) – 11p15.5 H19/ICR1 and KCNQ1OT1/ICR2. In normal physiology, H19/ICR1 is methylated on the paternal allele only and *IGF2* is expressed from this allele. In contrast, ICR2, located at the promoter region of *KCNQ1OT1* is methylated on the maternal allele only, leading to expression of *KCNQ1* and *CDKN1C* from the maternal allele. The normal physiologic imprinting status at 11p15.5 is referred to as 11p15.5 retention of imprinting (ROI) throughout this manuscript. Two predominant mechanisms occur that disrupt imprinting and result in biallelic expression of *IGF2*: 1) 11p15.5 H19/ICR1 loss of imprinting (LOI) refers to site-specific epigenetic gain of methylation at H19/ICR1 and 2) 11p15.5 copy neutral loss of heterozygosity (cn-LOH) refers to genetic deletion of the maternal allele and duplication of the paternal allele (paternal uniparental disomy). With 11p15.5 LOI, there is hypermethylation of H19/ICR1 and normal methylation of KCNQ1OT1/ICR2. With 11p15.5 LOH, there is hypermethylation of H19/ICR1 and hypomethylation of KCNQ1OT1/ICR2. Arrows indicate active transcription reflective of the normal physiologic imprinting pattern. Graphic made with biorender.com

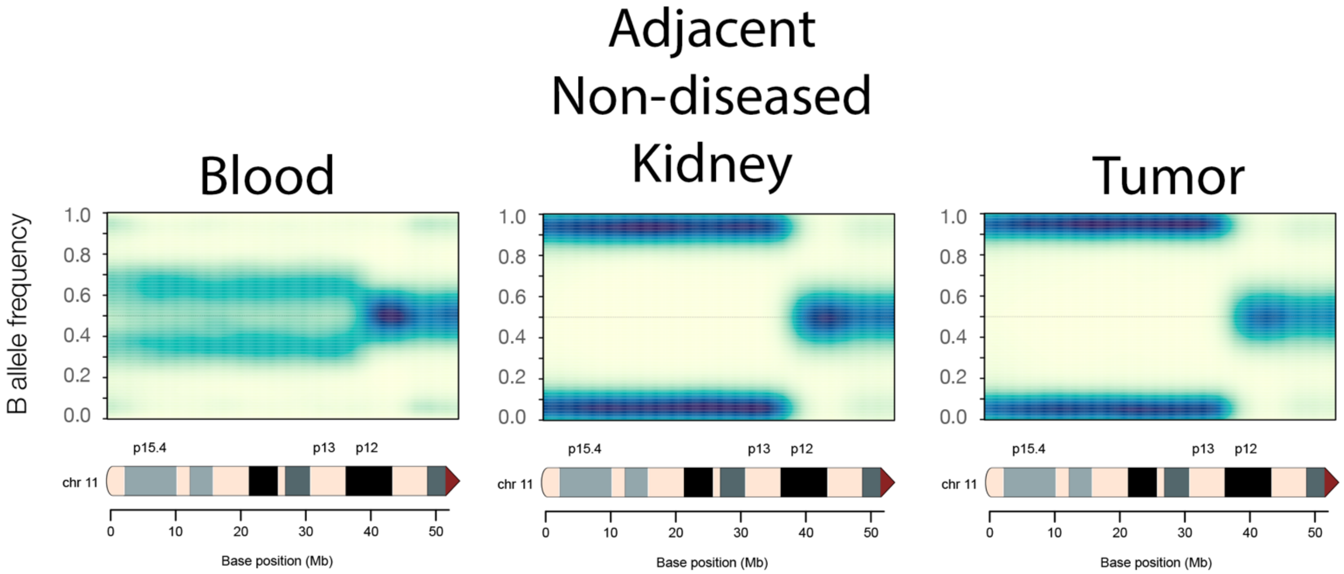

Supplementary Figure 2. Mosaicism for 11p15.5 loss of heterozygosity (paternal uniparental disomy) detected in DNA derived from peripheral blood leukocytes in patient SJWLM069390. A normal distribution of B allele frequency (both alleles detected at 0.5 is depicted starting at the 11p12 region and extending toward the chromosome 11 centromere. Complete 11p15.5 loss of heterozygosity (B allele frequency of 1.0) is observed in DNA derived from adjacent non-diseased kidney and Wilms tumor in the same patient. The region of 11p loss of heterozygosity in both the adjacent non diseased kidney and Wilms tumor overlaps the *IGF2* locus at 11p15.5 and the *WT1* locus at 11p13.

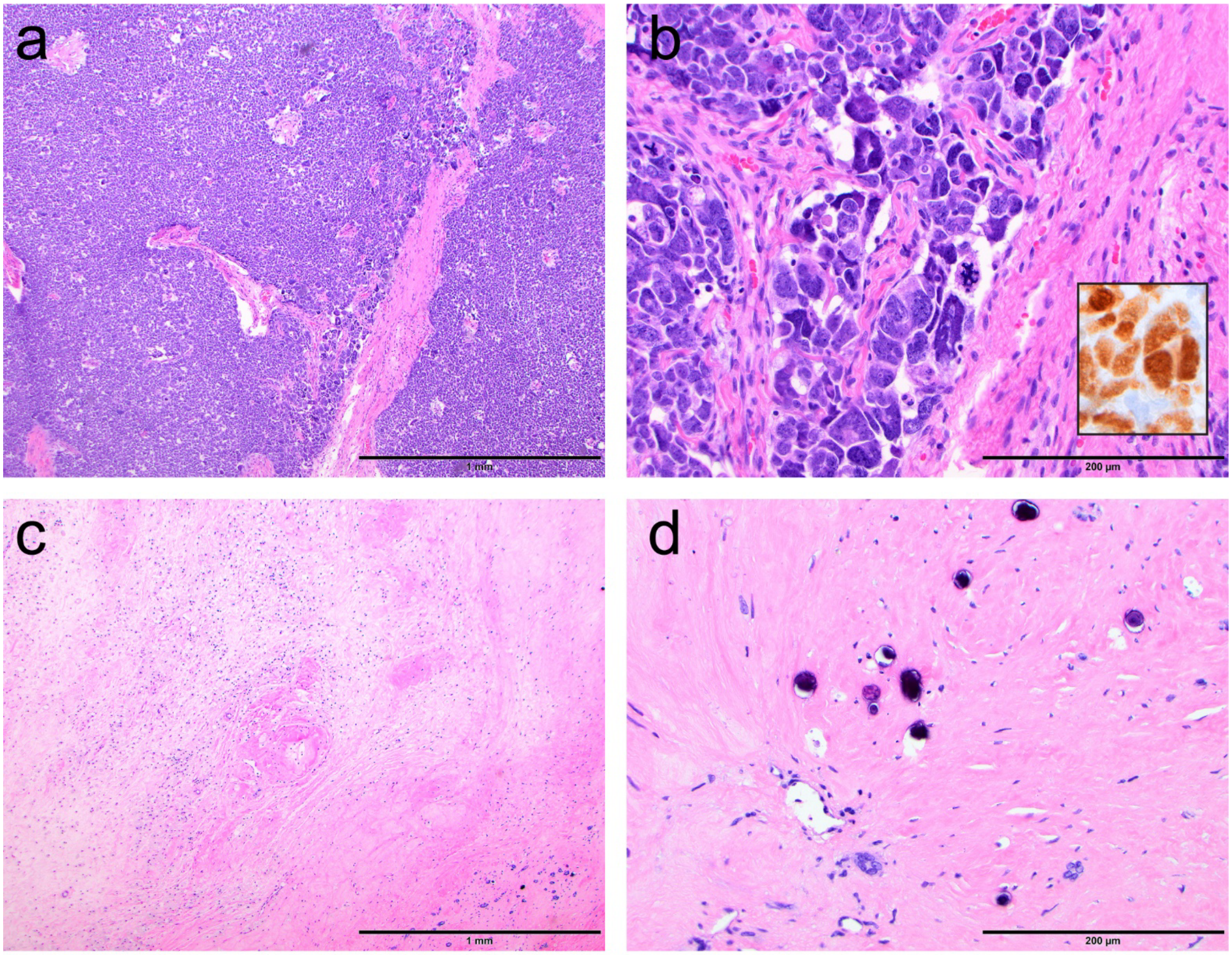

Supplementary Figure 3. Post-therapy treated synchronous bilateral Wilms tumors can exhibit markedly differential histology. A) Right lateral tumor, 4X H&E-stained section. Entirely viable tumor with diffuse anaplasia. Scale bar= 1mm B) Right lateral tumor, 20X H&E section showing diffuse anaplasia with atypical mitotic figures. p53 IHC stain (inset) showing diffuse positivity. Scale bar=200μm C) Left upper pole tumor, 4X H&E-stained section. Near total treatment response with fibrosis and inflammatory infiltration. Scale bar=1mm D) Left upper pole tumor, 20X H&E-stained section showing rare residual tubules and micro-calcifications. Scale bar= 200μm.

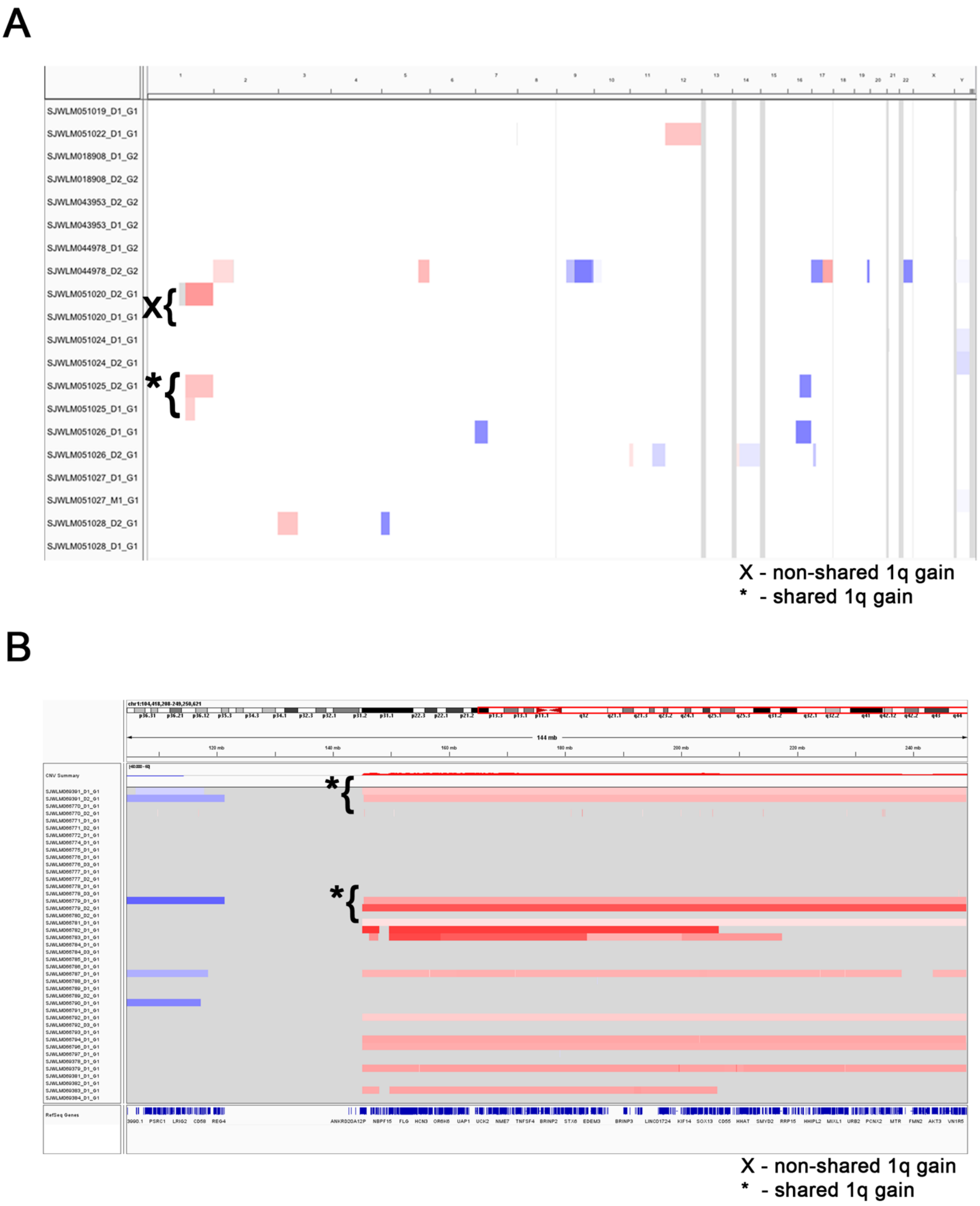

Supplementary Figure 4. 1q gain in paired synchronous bilateral Wilms tumors. 1q gain was shared in 3 of 4 synchronous BWT pairs (brackets with asterixis) for which any sample had 1q gain. Among these three synchronous BWT pairs with shared 1q gain, one pair exhibited differential extent of 1q gain (SJWLM051025, panel A) and two pairs (SJWLM069391 and SJWLM066779, panel B) exhibited gain of the entire 1q arm. The paired synchronous BWT SJWLM051020 exhibited one tumor with 1q gain and the contralateral tumor without (panel A, bracket with X). Panel A also depicts the genome-wide copy number profile of the paired synchronous BWT specimens from the St. Jude cohort. No pairs exhibited a similar genome-wide copy number profile.

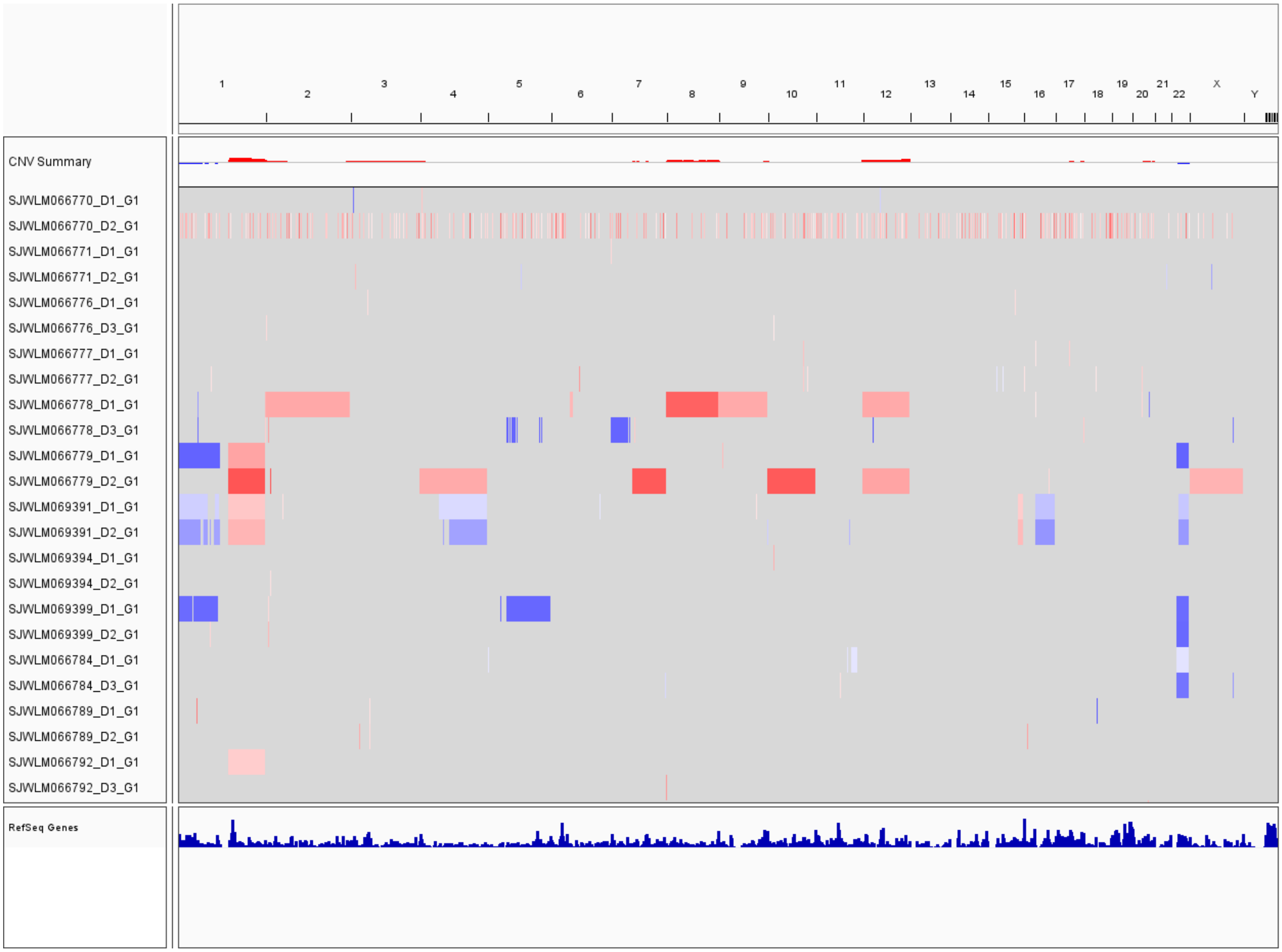

Supplementary Figure 5. Genome-wide copy number profiles differ in paired synchronous bilateral Wilms tumors. Rows on the x-axis demonstrate paired synchronous bilateral Wilms tumors (grouped next to one another) from the Children's Oncology Group cohort. Note that all pairs have dissimilar genome-side copy number profiles except for SJWLM069391. Synchronous bilateral Wilms tumor pairs from SJWLM069391, SJWLM069399, and SJWLM066784 exhibited similar loss of chromosome 22q.

A

B

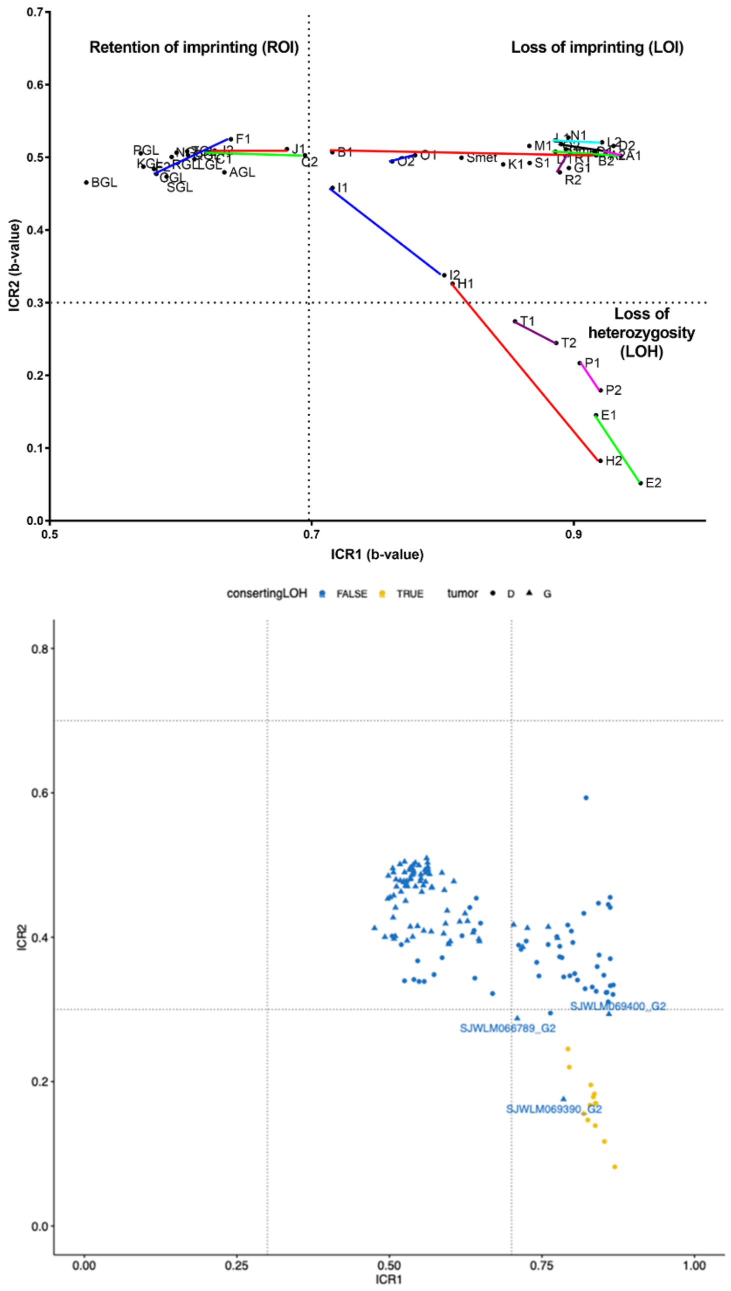

Supplementary Figure 6. Analysis of 11p15.5 imprinting status using methylation data. (A) Samples from SJCRH are depicted. Paired synchronous bilateral Wilms tumor samples are connected by colored lines. Samples marked with “GL” are germline DNA specimens derived from peripheral blood leukocytes and cluster in the 11p15.5 retention of imprinting quadrant. 11p15.5 imprinting status (ROI, LOI, LOH) was determined using methylation B values at 11p15.5 H19/ICR1 and ICR2 and status was shared in all except one case. N=11 biologically independent blood DNA samples and 34 tumor samples from COG cohort. (B) Validation of the methylation approach using whole genome sequencing of COG samples. Right lower quadrant samples colored in yellow demonstrate detection of 11p15.5 LOH using the CONSERTING algorithm. N=50 biologically independent blood DNA samples and 65 tumor samples from the COG cohort.

A

Sample	chrom	loc.start	loc.end	num.mark	LOH_seg.mean	CNV_LogRatio	CNV_Start	CNV_End	Type_Called
SJWLM051024_D1_G1	chr11	199193	47857253	658	0.451018237	NA	NA	NA	CnLoh
SJWLM051024_D2_G1	chr11	199193	47496827	641	0.462698908	NA	NA	NA	CnLoh
SJWLM051028_D1_G1	chr11	193202	45949201	601	0.408252912	NA	NA	NA	CnLoh
SJWLM051028_D2_G1	chr11	198062	36669778	576	0.431059028	NA	NA	NA	CnLoh

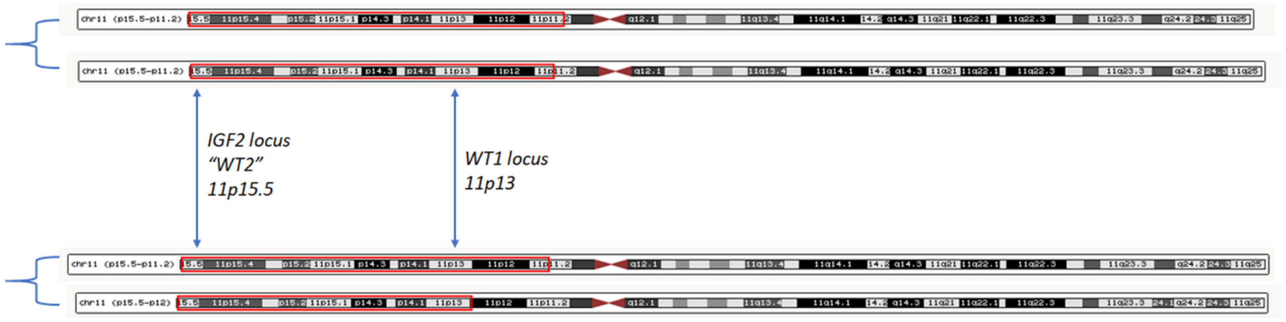

B

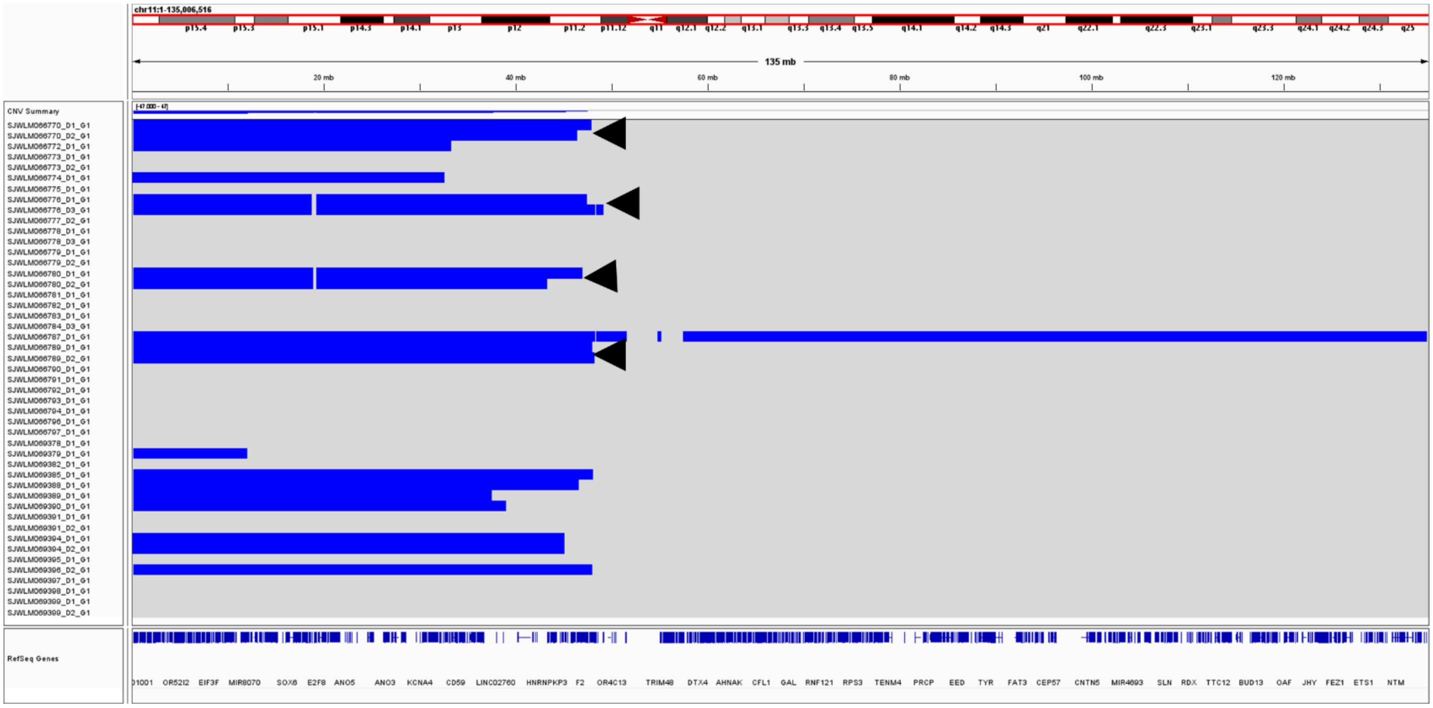

Supplementary Figure 7. (A) Paired synchronous bilateral Wilms tumors from the SJCRH dataset with pathogenic germline *WT1* variants, and shared 11p15.5 loss of heterozygosity illustrate that the area of 11p15.5 cnLOH overlaps both the 11p15.5 locus and the 11p13/*WT1* locus in extent. The extent of 11p LOH is different between paired synchronous bilateral Wilms tumor specimens as illustrated by the two sample pairs shown (SJWLM1024 and SJWLM1028). (B) Data derived from COG whole genome sequencing show differential extent of 11p LOH among paired synchronous bilateral pairs (4 examples shown with arrowheads), suggestive of independent genetic events that evolved in each tumor rather than a shared clonal origin. The extent of 11p LOH overlapped both the 11p15.5 and 11p13/WT1 locus in 18/19 cases.

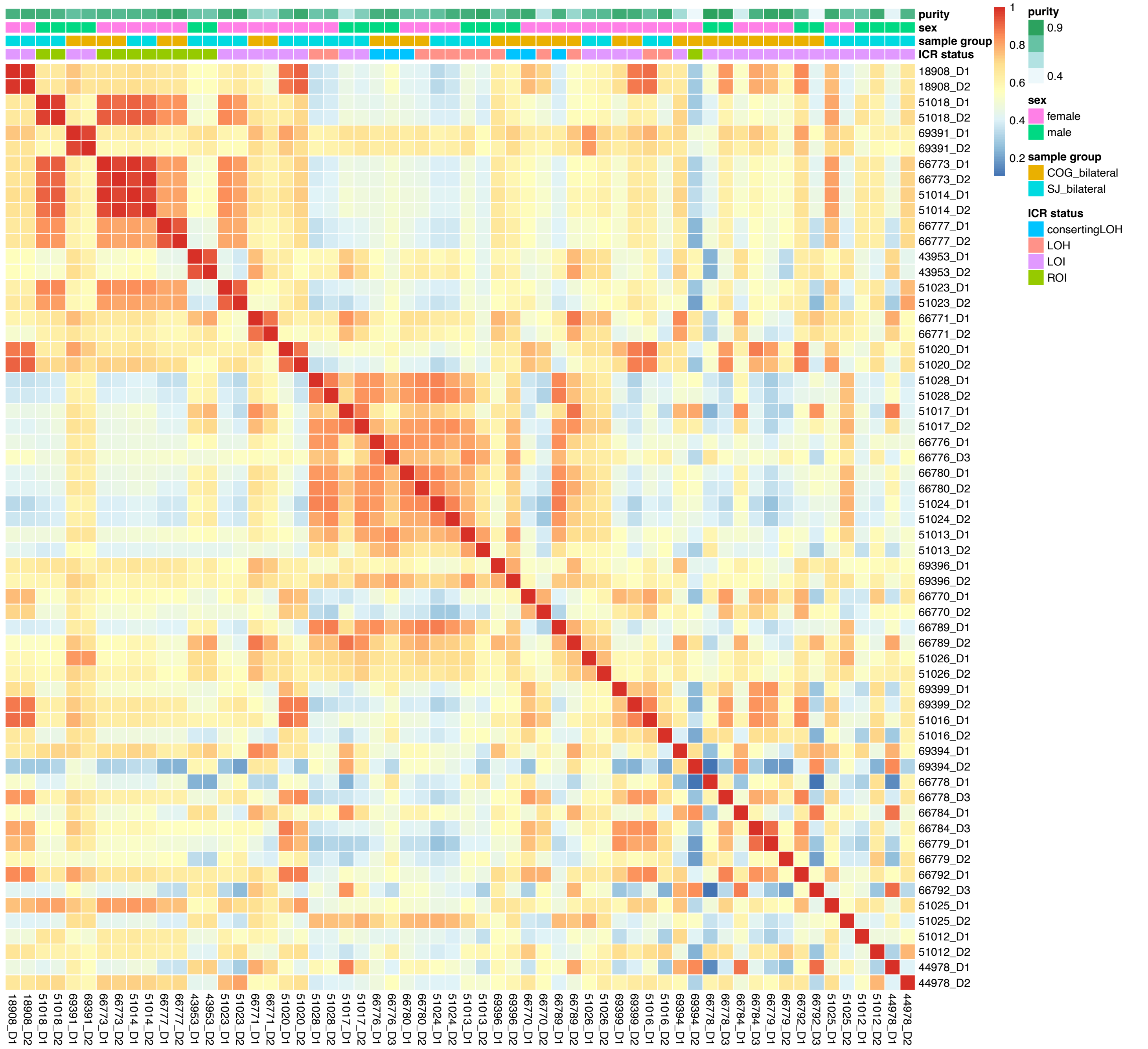

Supplementary Figure 8. A Spearman Correlation Matrix depicting pairs of synchronous bilateral Wilms tumor with correlation in methylation organized from greatest (left) to least (right). Tumor 11p15.5 status is depicted, which demonstrates a cluster of tumors with 11p15.5 loss of imprinting (LOI; purple) exhibiting the most inter-sample variability among paired synchronous bilateral Wilms tumors. N=60 total biologically independent paired synchronous bilateral tumor samples from 30 patients.

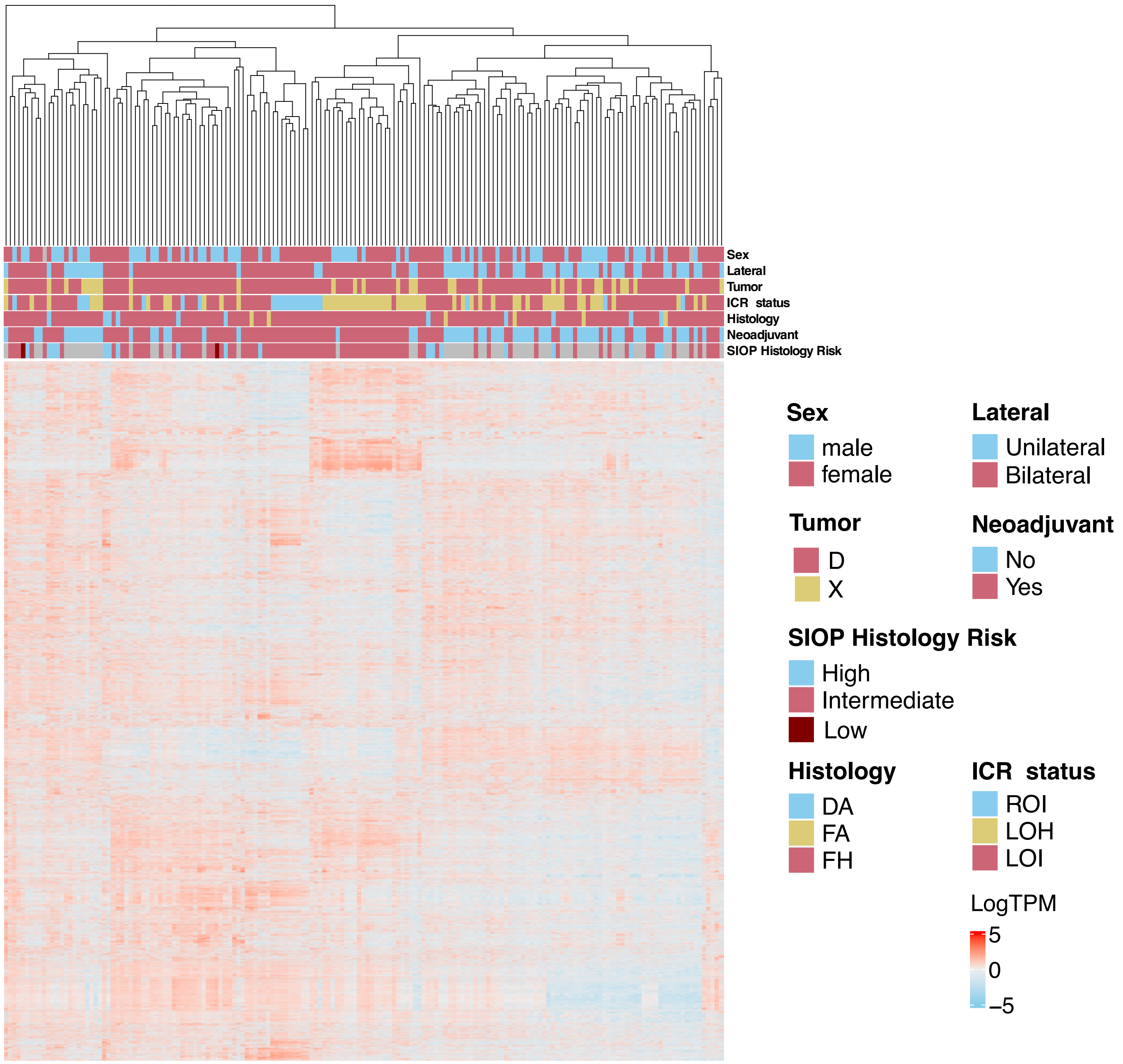

Supplementary Figure 9. Unsupervised hierarchical clustering of tumor total strand RNA-seq logTPM values. N=163 biologically independent samples.

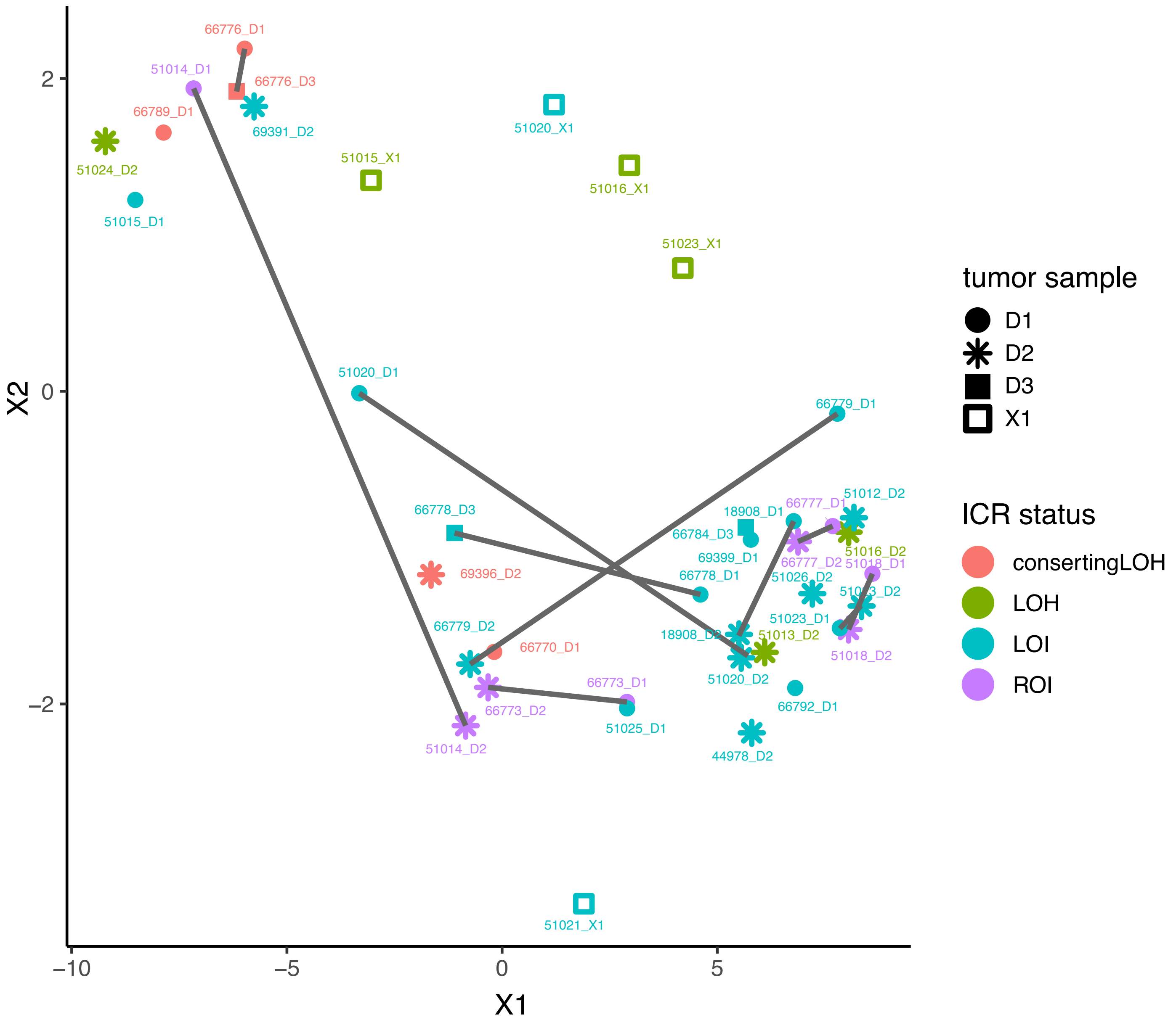

Supplementary Figure 10. TSNE clustering analysis of total strand RNA-seq log2 TPM values demonstrates less robust clustering of samples according to 11p15.5 status (LOH, LOI, ROI) and less robust clustering of paired synchronous bilateral Wilms tumor samples (grey lines connect samples) compared to TSNE clustering performed according to 850K EPIC Methylation Beadchip array data shown in main manuscript Figure 5. N=40 biologically independent samples.

Gene Name	Type	logFC (Bilateral-Unilateral)	P-Value	Adjusted P-Value
PPEF2	protein_coding	-2.176533241	7.07E-09	3.81E-05
METTL12	protein_coding	-1.442306213	1.40E-12	3.03E-08
SSTR5-AS1	processed_transcript	-1.273871276	6.27E-07	0.00112632
SNORD3C	lincRNA	-1.217415994	9.61E-08	0.00031741
SCARNA17	antisense	-1.146609211	1.09E-09	1.17E-05
SNORD3D	lincRNA	-1.141392927	2.57E-09	1.84E-05
RNU12	lincRNA	-1.128456629	1.18E-07	0.00031741
7SK	lincRNA	-1.106597589	5.82E-08	0.00025101
EIF4A1	protein_coding	-1.012750053	1.87E-07	0.00043002
SNORD3B-2	lincRNA	-0.988668468	1.09E-07	0.00031741
SNHG3	sense_intronic	-0.916016679	5.39E-07	0.00105589
RMRP	lincRNA	-0.793019041	1.99E-07	0.00043002

Supplementary Table 1. Differentially expressed genes comparing bilateral and unilateral Wilms tumor in this study. Twelve genes and/or noncoding RNAs were found to be upregulated in unilateral compared to bilateral WT. No genes were found to be upregulated in bilateral compared to unilateral WT. logFC – log fold change. Fisher exact test P-values are two-sided with adjustments for multiple comparisons shown (n=61 biologically independent samples).

Gene	Chr	P-value LOI vs ROI	Adjusted P-value LOI vs ROI	P-value LOH vs ROI	Adjusted P-value LOH vs ROI	LOH counts (N=14)	LOI counts (N=30)	ROI counts (N=13)	Fraction LOH	Fraction LOI	Fraction ROI
KCNQ1OT1	11	0.0753	0.543	3.39E-05	0.00464	0	29	10	0	0.967	0.769
KCNQ1	11	0.345	0.798	3.39E-05	0.00464	0	27	10	0	0.900	0.769
IGF2	11	0.000364	0.0462	0.00193	0.132	0	1	7	0	0.0333	0.538
INS-IGF2	11	0.000364	0.0462	0.00193	0.132	0	1	7	0	0.0333	0.538
H19	11	0.747	0.953	0.00580	0.318	0	16	6	0	0.533	0.462
SNORD116-4	15	0.00551	0.240	0.440	0.742	4	2	6	0.286	0.0667	0.462
RNF185	22	0.00384	0.240	0.0407	0.507	5	13	0	0.357	0.433	0
C1QL3	10	0.00662	0.240	0.0159	0.507	0	1	5	0	0.0333	0.385
CLEC12A	12	0.00579	0.240	0.0407	0.507	0	0	4	0	0	0.308
NPAS2	2	0.00579	0.240	0.164	0.618	1	0	4	0.0714	0	0.308

Supplementary Table 2. Cis-X integrated analysis of whole genome sequencing and total-strand RNA-seq data identifying genes with altered patterns of allele-specific expression according to 11p15.5 tumor methylation status in COG bilateral Wilms tumor samples. LOI – 11p15.5 H19/ICR1 loss of imprinting; LOH – 11p15.5 copy neutral heterozygosity/paternal uniparental disomy; ROI – 11p15.5 normal physiologic pattern of imprinting. Moderated t-test P-values are two-sided with adjustments for multiple comparisons shown (n=57 biologically independent samples).

SJWLM identifier	Chr 22 Somatic Copy Number Status	Chr 11p15.5 somatic imprinting status	RNF185 reduction to allele specific expression	RNF185 LOH/ Del	DGCR8 E518K somatic/tumor	SIOP Histology Risk
066779	LOH right<br>Normal left	LOI	Yes	LOH	Yes	High
066784	LOH (subclonal) right<br>LOH left	LOI	Yes	Yes	Yes	Intermediate
066787	LOH (subclonal)	LOI	No	Low level LOH	No	Intermediate
066790	LOH	LOI	Yes	LOH	Yes	High
066796	LOH	LOI	N/A	N/A	No	High
069391	LOH (subclonal) right<br>LOH left	LOI	No	Low level LOH	No	N/A
044978	Normal right<br>LOH left	LOI	N/A	N/A	No	High
069399	LOH right<br>LOH left	LOI	Yes	LOH	Yes	High

Supplementary Table 3. Molecular and clinical characteristics of bilateral Wilms tumor patients with any tumor containing copy number loss of chromosome 22q.
