# Supplementary material for: Genetic and epigenetic features of bilateral Wilms tumor predisposition in patients from the Children’s Oncology Group AREN18B5-Q
Source: Nat Commun. 2023 Dec 18;14:8006. doi: 10.1038/s41467-023-43730-0 (PMC10728430; doi:10.1038/s41467-023-43730-0)
Supplement: Supplementary file 3 — Description of Additional Supplementary Files [file 41467_2023_43730_MOESM3_ESM.pdf]

Description of Additional Supplementary Files

File Name: Supplementary Data 1
Description: Germline genetic variants from peripheral blood DNA in St. Jude and COG bilateral Wilms tumor patients.

File Name: Supplementary Data 2
Description: Somatic tumor genetic variants in St. Jude and COG bilateral Wilms tumor samples.

File Name: Supplementary Data 3
Description: DAVID pathway analysis of somatic tumor genetic variants.

File Name: Supplementary Data 4
Description: Genetic variants detected in adjacent nondiseased kidney tissue in the COG bilateral Wilms tumor cohort.

File Name: Supplementary Data 5
Description: Methylation Beta values at 11p15.5 H19/ICR1 and ICR2 in tumor, blood, and adjacent kidney samples
