# Supplementary material for: Genetic and epigenetic features of bilateral Wilms tumor predisposition in patients from the Children’s Oncology Group AREN18B5-Q
Source: Nat Commun. 2023 Dec 18;14:8006. doi: 10.1038/s41467-023-43730-0 (PMC10728430; doi:10.1038/s41467-023-43730-0)
Supplement: Supplementary file 9 — Reporting Summary [file 41467_2023_43730_MOESM9_ESM.pdf]

nature portfolio

Corresponding author(s): Andrew J. Murphy, MD
Xiang Chen, PhD

Last updated by author(s): Nov 7, 2023

Reporting Summary

Nature Portfolio wishes to improve the reproducibility of the work that we publish. This form provides structure for consistency and transparency in reporting. For further information on Nature Portfolio policies, see our Editorial Policies and the Editorial Policy Checklist.

Statistics

For all statistical analyses, confirm that the following items are present in the figure legend, table legend, main text, or Methods section.

n/a	Confirmed	
☐	☒	The exact sample size (*n*) for each experimental group/condition, given as a discrete number and unit of measurement
☐	☒	A statement on whether measurements were taken from distinct samples or whether the same sample was measured repeatedly
☐	☒	The statistical test(s) used AND whether they are one- or two-sided<br>*Only common tests should be described solely by name; describe more complex techniques in the Methods section.*
☒	☐	A description of all covariates tested
☐	☒	A description of any assumptions or corrections, such as tests of normality and adjustment for multiple comparisons
☐	☒	A full description of the statistical parameters including central tendency (e.g. means) or other basic estimates (e.g. regression coefficient) AND variation (e.g. standard deviation) or associated estimates of uncertainty (e.g. confidence intervals)
☐	☒	For null hypothesis testing, the test statistic (e.g. *F*, *t*, *r*) with confidence intervals, effect sizes, degrees of freedom and *P* value noted<br>*Give P values as exact values whenever suitable.*
☒	☐	For Bayesian analysis, information on the choice of priors and Markov chain Monte Carlo settings
☐	☒	For hierarchical and complex designs, identification of the appropriate level for tests and full reporting of outcomes
☐	☒	Estimates of effect sizes (e.g. Cohen's *d*, Pearson's *r*), indicating how they were calculated

Our web collection on statistics for biologists contains articles on many of the points above.

Software and code

Policy information about availability of computer code

Data collection	See below Data analysis for complete list of software.
Data analysis	Adobe Photoshop v23.2.2 (Adobe, San Jose, CA)<br><br>Microsoft Excel v16.78.3 (Microsoft, Redmond, WA)<br><br>Microsoft Word v16.78.3 (Microsoft, Redmond, WA)<br><br>GraphPad Prism version v10 (GraphPad Software, La Jolla, CA)<br><br>R Software version 3.5.1 and 4.1.0 (www.rproject.org) using the following packages:<br>--edgeR Bioconductor package (https://bioconductor.org/packages/release/bioc/html/edgeR.html)<br>--limma Bioconductor package (https://bioconductor.org/packages/release/bioc/html/limma.html)<br>--DNAcopy Bioconductor package (https://bioconductor.org/packages/release/bioc/html/DNAcopy.html)<br>--Minifi Bioconductor package (https://bioconductor.org/packages/release/bioc/html/minfi.html)<br>--HTseq-count (https://htseq.readthedocs.io/en/release_0.11.1/count.html)<br><br>Python v2.7.2 (https://www.python.org/download/releases/2.7.2/); (Python Software Foundation, Wilmington, DE) using the following packages:<br>--SciPy (https://www.scipy.org/)

--Pandas (https://pandas.pydata.org/)
--NumPy (http://www.numpy.org/)
--Scikit-learn (https://scikit-learn.org/stable/)
--Seaborn (https://seaborn.pydata.org/)

Integrated Genomics Viewer (IGV) v2.10.2 (Broad Institute, Cambridge, MA)

DAVID Functional Annotation Tool (Database for Annotation, Visualization, and Integrated Discovery; https://david.ncifcrf.gov/)

CONSERTING algorithm (Copy Number Segmentation by Regression Tree in Next Generation Sequencing; https://www.stjude.org/research/labs/zhang-lab/conserting.html)

Cis-X algorithm (https://www.stjude.org/research/labs/zhang-lab/cis-x.html)

Biorender App (https://www.biorender.com/)

Olympus cellSens Standard v1.18 (Build 16686) (Olympus, Tokyo, Japan)

Clinvar database (https://www.ncbi.nlm.nih.gov/clinvar/)

PROVEAN (Protein Variation Effect Analyzer) algorithm (https://www.jcvi.org/research/provean)

Polyphen2 algorithm (http://genetics.bwh.harvard.edu/pph2/)

No custom code utilized

For manuscripts utilizing custom algorithms or software that are central to the research but not yet described in published literature, software must be made available to editors and reviewers. We strongly encourage code deposition in a community repository (e.g. GitHub). See the Nature Portfolio guidelines for submitting code & software for further information.

Data

Policy information about availability of data

All manuscripts must include a data availability statement. This statement should provide the following information, where applicable:

- Accession codes, unique identifiers, or web links for publicly available datasets
- A description of any restrictions on data availability
- For clinical datasets or third party data, please ensure that the statement adheres to our policy

Data Availability:
1. The 850K MethylationEPIC and RNA-seq data from the St. Jude and Children's Oncology Group bilateral Wilms tumor patients generated in this study are uploaded to the Gene Expression Omnibus (GEO) database (https://www.ncbi.nlm.nih.gov/geo/) under SuperSeries accession number GSE226480 (https://www.ncbi.nlm.nih.gov/geo/query/acc.cgi?acc=GSE226480). The Whole exome sequencing and whole genome sequencing data generated in this study are available at the NCBI Sequence Read Archive (SRA) database under accession number PRJNA943166 (https://www.ncbi.nlm.nih.gov/bioproject/PRJNA943166).

2. 850K MethylationEPIC data used from our prior publication (Murphy, et. al. Nat Commun. 2019 Dec 20;10(1):5806. doi: 10.1038/s41467-019-13646-9) which are used for comparative analysis in the current study are available via the GEO database under accession number GSE110697 (https://www.ncbi.nlm.nih.gov/geo/query/acc.cgi?acc=GSE110697).

3. 850K MethylationEPIC data from the Wilms tumor survivorship cohort and healthy community controls (Song, et. al. Genome Med. 2021 Apr 6;13(1):53. doi: 10.1186/s13073-021-00875-1) are available via the GEO database under accession numbers GSE197676 (https://www.ncbi.nlm.nih.gov/geo/query/acc.cgi?acc=GSE197676), GSE197675 (https://www.ncbi.nlm.nih.gov/geo/query/acc.cgi?acc=GSE197675), and GSE197674 (https://www.ncbi.nlm.nih.gov/geo/query/acc.cgi?acc=GSE197674).

4. Source Data are provided as a Source Data File. The remaining data are available within the Article, Supplementary Information, or Source Data File.

Research involving human participants, their data, or biological material

Policy information about studies with human participants or human data. See also policy information about sex, gender (identity/presentation), and sexual orientation and race, ethnicity and racism.

Reporting on sex and gender	The term biological sex is used throughout the manuscript. Gender identity was not assessed. The biological sex of study subjects was self-reported and also verified using whole genome sequencing and 850K methylationEPIC beadchip data. Data are reported according to biological sex in Figure 2 (heatmap summary of findings in study). In addition, data are reported according to biological sex in the manuscript text for results of the exploratory analysis looking at 11p15.5 H19/ICR1 low-level hypermethylation detectable in peripheral blood because the majority of such patients meeting these criteria were female.
Reporting on race, ethnicity, or other socially relevant groupings	Race,ethnicity, and other socially relevant groupings are not utilized in this study.

Population characteristics	All samples utilized in this study are from pediatric patients between two months and 18 years of age. All included patients had a diagnosis of synchronous bilateral Wilms tumor.
Recruitment	Patient samples included in this study were retrospectively identified from the St. Jude Children's Research Hospital Biorepository (https://www.stjude.org/research/why-st-jude/shared-resources/biorepository.html) or from the Children's Oncology GroupBiopathology Center (https://childrensoncologygroup.org/obtainingbiospecimens). Children's Oncology Group specimens were from patients enrolled on AREN0534 (Treatment for Patients with Bilateral, Multicentric, or Bilaterally-Predisposed Unilateral Wilms Tumor) or AREN03B2 (Renal Tumors Classification, Biology, and Banking Study). Although consents allowedfor the research conducted in this study in all cases, patients were not specifically recruited to this biology study.
Ethics oversight	Children's Oncology Group and NCI-Clinical Trials Evaluation Program (COG AREN18B5-Q). St. Jude Children's Research Hospital Institutional Review Board (IRB# Pro00007515).

Note that full information on the approval of the study protocol must also be provided in the manuscript.

Field-specific reporting

Please select the one below that is the best fit for your research. If you are not sure, read the appropriate sections before making your selection.

☒ Life sciences ☐ Behavioural & social sciences ☐ Ecological, evolutionary & environmental sciences

For a reference copy of the document with all sections, see nature.com/documents/nr-reporting-summary-flat.pdf

Life sciences study design

All studies must disclose on these points even when the disclosure is negative.

Sample size	No statistical method was utilized to pre-determine sample size. Given the rare nature of bilateral Wilms tumor, this study was designed to maximize the number of eligible paired synchronous bilateral Wilms tumor specimens.
Data exclusions	Samples from two patients initially provided by the Children's Oncology Group were excluded from analysis because the tumor samples did not originate from the same individual as the provided germline samples (germline/tumor mismatch).
Replication	An initial cohort consisting of samples from 18 total patients with bilateral Wilms tumor from St. Jude Children's Research Hospital was first independently analyzed. Preliminary findings from this discovery analysis were utilized to justify application for additional specimens from the Children's Oncology Group. An expansion cohort of 50 additional patients with bilateral Wilms tumor was assembled from the Children's Oncology Group. The finding of 11p15.5 mosaicism was validated using an additional dataset from the St. Jude Life cohort (described in manuscript and data availability statement) consisting of DNA from 282 healthy community controls, 68 BWT patients and long-term survivors, and 146 unilateral WT patients and long-term survivors. Key findings from the initial St. Jude cohort were replicated in the Children's Oncology Group cohort and are outlined in Figure 2 of the manuscript.
Randomization	Randomization is not relevant to this study because it did not involve clinical treatments or experimental interventions to living organisms.
Blinding	Investigators were blinded to reported clinical details including patient biological sex, phenotype consistent with congenital syndrome, tumor histology, and presence of nephrogenic rests until after molecular data were determined and analyzed.

Reporting for specific materials, systems and methods

We require information from authors about some types of materials, experimental systems and methods used in many studies. Here, indicate whether each material, system or method listed is relevant to your study. If you are not sure if a list item applies to your research, read the appropriate section before selecting a response.

Materials & experimental systems

n/a	Involved in the study	
☒	☐	Antibodies
☒	☐	Eukaryotic cell lines
☒	☐	Palaeontology and archaeology
☒	☐	Animals and other organisms
☐	☒	Clinical data
☒	☐	Dual use research of concern
☒	☐	Plants

Methods

n/a	Involved in the study	
☒	☐	ChIP-seq
☒	☐	Flow cytometry
☒	☐	MRI-based neuroimaging

Clinical data

Policy information about clinical studies

All manuscripts should comply with the ICMJE guidelines for publication of clinical research and a completed CONSORT checklist must be included with all submissions.

Clinical trial registration	Children's Oncology Group AREN18B5-Q: Genomic Analysis of Bilateral Wilms Tumor
Study protocol	Children's Oncology Group Biology Study - no clinical trial protocol.
Data collection	Molecular data were collected and analyzed between 2017 (St. Jude) or 2018 (COG) and 2023.
Outcomes	There were no primary or secondary clinical outcomes associated with this biology-based study. This is a translational biospecimens study that does not examine clinical outcomes and therefore the CONSORT checklist is not applicable to this research design.

Plants

Seed stocks	*Report on the source of all seed stocks or other plant material used. If applicable, state the seed stock centre and catalogue number. If plant specimens were collected from the field, describe the collection location, date and sampling procedures.*
Novel plant genotypes	*Describe the methods by which all novel plant genotypes were produced. This includes those generated by transgenic approaches, gene editing, chemical/radiation-based mutagenesis and hybridization. For transgenic lines, describe the transformation method, the number of independent lines analyzed and the generation upon which experiments were performed. For gene-edited lines, describe the editor used, the endogenous sequence targeted for editing, the targeting guide RNA sequence (if applicable) and how the editor was applied.*
Authentication	*Describe any authentication procedures for each seed stock used or novel genotype generated. Describe any experiments used to assess the effect of a mutation and, where applicable, how potential secondary effects (e.g. second site T-DNA insertions, mosiacism, off-target gene editing) were examined.*
